# Effectiveness of Protected Areas for Representing Species and Populations of Terrestrial Mammals in Costa Rica

**DOI:** 10.1371/journal.pone.0124480

**Published:** 2015-05-13

**Authors:** José F. González-Maya, Luis R. Víquez-R, Jerrold L. Belant, Gerardo Ceballos

**Affiliations:** 1 Instituto de Ecología, Universidad Nacional Autónoma de México, Ciudad Universitaria, 04318, México D. F., México; 2 Proyecto de Conservación de Aguas y Tierras, ProCAT Colombia/Internacional, Carrera 13 No. 96-82 Of. 205, Bogotá, Colombia; 3 Carnivore Ecology Laboratory, Forest and Wildlife Research Center, Mississippi State University, 39759, Starkville, Mississippi, United States of America; Instituto de Pesquisas Ecológicas, BRAZIL

## Abstract

Costa Rica has one of the greatest percentages (26%) of protected land in the world. The National Protected Areas System (NPAS) of Costa Rica was established in 1976 and currently includes >190 protected areas within seven different protection categories. The effectiveness of the NPAS to represent species, populations, and areas with high species richness has not been properly evaluated. Such evaluations are fundamental to understand what is necessary to strengthen the NPAS and better protect biodiversity. We present a novel assessment of NPAS effectiveness in protecting mammal species. We compiled the geographical ranges of all terrestrial Costa Rican mammals then determined species lists for all protected areas and the estimated proportion of each species’ geographic range protected. We also classified mammal species according to their conservation status using the IUCN Red List of Threatened Species. We found almost complete representation of mammal species (98.5%) in protected areas, but low relative coverage (28.3% on average) of their geographic ranges in Costa Rica and 25% of the species were classified as underprotected according to a priori representation targets. Interestingly, many species-rich areas are not protected, and at least 43% of cells covering the entire country are not included in protected areas. Though protected areas in Costa Rica represent species richness well, strategic planning for future protected areas to improve species complementarity and range protection is necessary. Our results can help to define sites where new protected areas can have a greater impact on mammal conservation, both in terms of species richness and range protection.

## Introduction

Protected areas are the primary biodiversity conservation strategy used across geographic scales to avert biodiversity loss [1, 2]. Though global biodiversity conservation goals for protected areas have been set to reduce possible negative effects of anthropogenic activities [3], many aspects of protected areas, such as representativeness of species and ecosystems, have not been fully examined [2, 4]. Though the global biodiversity crisis is typically measured at the species level, the effects of species loss occurs first at the population level [5]. Because of the need for understanding conservation strategies at finer scales, the role of protected areas has gained greater relevance and their importance requires further assessment [6]. As more and better information is available regarding species distributions and population trends, assessments of protected area effectiveness can be conducted at increasingly finer resolutions [7]. Additionally, as new data becomes available, the use of global range data derived from systematic efforts can further help improve conservation effectiveness assessments.

Costa Rica’s protected areas system is considered among the most successful in Latin America [8]. Established in 1976, Costa Rica’s National Protected Areas System (NPAS) has evolved from a few areas of small geographic extent to a large and well-managed system [8, 9]. However, the full extent of protected areas effectiveness in representing species and other aspects of biodiversity conservation in Costa Rica have not been addressed. Further, the Convention for Biological Diversity goals [10] and earlier assessments have identified substantive under-representation of numerous ecosystems within this protected area network [11]. Given the need for assessing effectiveness at various biodiversity levels, and that most countries lack comprehensive information for many taxonomic groups, surrogate and/or particular taxonomic groups and their conservation status have been used as indicators of protected area effectiveness [12, 13]. Mammals play key roles in ecosystems, are considered charismatic and require relatively large areas to survive, therefore their conservation is warranted [4, 5, 14]. Furthermore, there is often more data available on mammals than for other taxonomic groups [15, 16]; therefore, we consider mammals an appropriate group for assessing representativeness of protected areas. We present the first comprehensive assessment of Costa Rican protected areas effectiveness and representativeness using the most recent geographic information for protected areas and the most updated available range information for mammals. Our objectives were to: 1) evaluate the extent and representativeness of protected areas for protecting terrestrial mammal species and their geographic ranges in Costa Rica, 2) determine priority areas for mammal conservation, and 3) assess the singularities and conservation gaps for mammal species within the NPAS.

## Materials and Methods

### Study area

Costa Rica is located in Mesoamerica between 8° and 12° N and 82° and 86° W, bordering with Nicaragua and Panama to the north and south, respectively. It has 51,100 km^2^ of continental territory. Considered one of the most biologically diverse countries in the world [17], Costa Rica has strong environmental policies and a long-standing NPAS system [18], with more than 190 protected areas covering about 26% of the national territory [19, 20]. Due to its geographical position, Costa Rica has played a key role in the Great Continental Interchange [21, 22] and has a mixture of Neotropical and Neartic faunas [23–25]. Costa Rica has nearly 238 mammal species, including >200 terrestrial species within 140 genera and 44 families [24, 26].

### Data sources

Species geographic distributions were obtained from the 2008 Global Mammal Assessment [27] available through the IUCN Red List of Threatened Species (S1 and S2 Tables; [27, 28]). Each range was delineated, assessed and revised by a group of mammalogists from the respective countries where the species are present [27] and represented the best information available. We classified each species as ‘native’, ‘extant’ and ‘possibly extant’, checking for consistency with the most recent species list for the country [24]. We also obtained the conservation status of each species [28], which included Critically Endangered (CR), Endangered (EN), Vulnerable (VU), Near Threatened (NT), Least Concern (LC) and Data Deficient (DD). Finally, we identified endemic species (*n* = 18), defined as those unique to Costa Rica or distributed only in Costa Rica-Panama or Costa Rica-Nicaragua [24, 26]. Taxonomic changes that occurred since the last Costa Rican mammal list update were considered for our analyses (S1 Text).

We obtained spatial information regarding protected areas from the Sistema Nacional de Áreas de Conservación—SINAC (Conservation Areas National System), the most current database for protected areas in Costa Rica that contained sub-national level areas not included in the World Database for Protected Areas [29]. We used only those protected areas (*n* = 198) with an IUCN Protected Areas management category [29, 30] (Table 1). Based on land cover data for the country [31], we estimated that 88.03% of protected areas consist of natural ecosystems, with 11.56% considered non-forested areas (secondary growth and regeneration), and 0.39% classified as agricultural and urban areas.

**Table 1:** Protected areas of Costa Rica classified by IUCN categories, its corresponding national category, number of areas in each category and area covered for effectiveness assessment for mammal conservation.

IUCN Category	Costa Rica Category	Abb.	Number of areas	Area (km^2^)
I	Biological Reserves	BR	8	216.0
II	Absolute Natural Reserve	ANR	1	13.0
	National Park	NP	32	6274.8
IV	Wildlife Refuges	WR	90	2338.7
	National Wetland	NT	21	302.0
VI	Forestry Reserve	FR	11	2166.2
	Protected Zone	PZ	35	1575.0

### Effectiveness of protected areas in representing species richness

To assess the effectiveness of protected areas for protecting total species richness, we determined the complementarity and representativeness of all mammal species in the protected areas system. We defined complementarity as the gain in representativeness of mammal species when a site is added to an existing set of protected areas [32, 33]. We overlaid the protected areas map with the distribution polygons of all 208 species and extracted the number and identity of each species for each protected area by protected area category. We then sorted the protected areas by year of establishment using the SINAC database and generated a species accumulation curve. We assessed complementarity, high or low redundancy, by generating an accumulation curve for the randomized pooled protected areas and assessed similarities in species composition (i.e. species identity) among protected areas and categories through a cluster analysis using the Jaccard similarity coefficient; Jaccard coefficients measures spatial turnover by comparing all pairs sites, clustering similar sites until a complete dendrogram is constructed [34, 35]. Considering the variation in size among protected areas, we tested for the relationship between the extent of each protected area and the number of species protected using a simple linear regression.

### Effectiveness of protected areas for protecting mammal species ranges

We used a digital vector map of Costa Rica to determine the geographic range of each species within its boundaries [31]. We then overlaid the range polygons of the 208 mammal species with the protected areas polygons and estimated the proportion of each species’ range currently within the protected areas by category. We assessed the progression of species’ range protection according to year of establishment by chronologically ordering the range proportion of each species added per area created each year. We used simple Pearson correlation tests to assess relationships between the geographic extent of each category and the mean range protected and to test if the proportion of range protected was associated with total species range.

To assess if a species is “underprotected” (i.e., if it has an insufficient coverage of its geographic range), we followed the targets proposed by Rodrigues et al. [36] but adapted to Costa Rica: restricted-range species (<1000 km2) were expected to be 100% protected while large range species (>25,550 km^2^, half the country´s total extent) were expected to be protected in at least 10% of their range, and a linear decline in the target between these two extremes [37]. We plotted the current range for each species, the proportion of its range protected and the a priori representation targets and identified as underprotected those species below this target.

### Conservation gaps and priority areas

To determine conservation gaps and priorities we overlaid all species distribution polygons with a grid containing 83 km^2^ cells and from each cell we extracted the species present in each cell, evaluating the total number of species and the number of species on each IUCN Red List category. We defined our mapping unit as this cell size considering the mean range of the five smallest species ranges in the country, thus ensuring that each of these species could be considered in at least one cell. We overlaid this grid with the protected areas digital layer and identified those cells with number of species and threatened species (i.e., Critically Endangered, Endangered, Vulnerable and Data Deficient) not covered by any protected area as well as those areas with the highest numbers of underprotected species according to the a priori targets. We included Data Deficient species among those threatened since this category represents those species with not sufficient information to be properly assessed but acknowledges the possibility that future research will indicate that a threatened category may be appropriate [38]. We considered a cell to be protected when it was partially overlapping with at least one protected area. We tested for differences (α = 0.05) in total species richness and number of endemic and threatened species between protected and unprotected cells using non-parametric Kruskal Wallis tests and Conover post-hoc multiple comparison tests [39]. All statistical analyses were performed using Infostat software [39] and all spatial analyses with ArcGIS 10.2 [40]. Means are reported with ± 1 SD unless otherwise noted.

## Results

### Representativeness of mammal species and richness in protected areas

Most species (98.5%; 205 spp.) are represented in the NPAS. Only three species, *Coendou rothschildi*, *Peromyscus gymnotis* and *Reithrodonthomys paradoxus*, do not have any portion of their range protected. Of the 18 endemic species, all occur within at least three protected areas (Mean±SD = 25.72 ± 16.41), with *Sturnira mordax* and *Cryptotis nigrescens* the best represented (59 and 55 protected areas, respectively) and *Orthogeomys cavator* and *Sigmodontomys aphrastus* the poorest represented (3 and 4 protected areas, respectively; Table 2). Three protected areas contained 12 endemic species (Cordillera Volcánica Central Forestry Reserve and La Amistad and Chirripó National Parks). For threatened species, the mean number of protected areas for all species is greater for Endangered (153.00 ± 0.00), than Data Deficient (73.43 ± 75.40) and Vulnerable (69.35 ± 56.00) species. La Amistad and Chirripó National Parks and Los Santos Forestry Reserve each harbor 11 threatened species. *Leopardus tigrinus*, C*ryptotis gracilis* and *Saimiri oerstedii* (VU) represented in 48, 39 and 37 protected areas, respectively, and *Cryptotis orophila*, *Sylvilagus dicei* and *Sigmodontomys aphrastus* (DD) represented in 38, 13 and 4 protected areas, respectively, are the least represented of all threatened species.

**Table 2:** Endemic and threatened species representativeness and range protected in the protected areas system of Costa Rica.

Species	Range protected (%)	Protected Areas	Endemics			Red List status				
			CR	CR-P	CR-N	EN	VU	DD	NT	LC
*Reithrodontomys paradoxus*	0.00	0						*RL*		
*Orthogeomys cherriei*	4.88	22	E							*RL*
*Orthogeomys heterodus*	9.13	11	E							*RL*
*Reithrodontomys brevirostris*	12.31	28			E					*RL*
*Mazama temama*	19.21	153						*RL*		
*Ateles geoffroyi*	19.26	153				*RL*				
*Tapirus bairdii*	19.26	153				*RL*				
*Cabassous centralis*	19.26	153						*RL*		
*Lontra longicaudis*	19.26	153						*RL*		
*Myrmecophaga tridactyla*	19.63	153					*RL*			
*Cryptotis orophila*	23.68	38						*RL*		
*Sturnira mordax*	24.90	59		E					*RL*	
*Saimiri oerstedii*	25.54	37					*RL*			
*Orthogeomys underwoodi*	27.65	36	E							*RL*
*Cryptotis nigrescens*	32.39	55		E						*RL*
*Reithrodontomys rodriguezi*	34.18	14	E							*RL*
*Leopardus tigrinus*	35.98	48					*RL*			
*Cryptotis gracilis*	37.63	39		E			*RL*			
*Heteromys oresterus*	40.08	9	E							*RL*
*Reithrodontomys creper*	41.76	34		E						*RL*
*Rheomys raptor*	43.63	39		E						*RL*
*Scotinomys xerampelinus*	51.16	16		E						*RL*
*Syntheosciurus brochus*	57.22	26		E					*RL*	
*Sylvilagus dicei*	61.57	13		E				*RL*		
*Sigmodontomys aphrastus*	61.83	4		E				*RL*		
*Orthogeomys cavator*	62.06	3		E						*RL*
*Rheomys underwoodi*	68.44	18		E						*RL*

Endemic species to Costa Rica (CR), to Costa Rica-Panama (CR-P) and to Costa Rica-Nicaragua (CR-N) are indicated by E. Species classified according to the IUCN Red List Criteria [28] as Endangered (EN), Vulnerable (VU), Data Deficient (DD), Near Threatened (NT) and Least Concern (LC) are indicated by *RL* (Red List).

**Fig 1:**
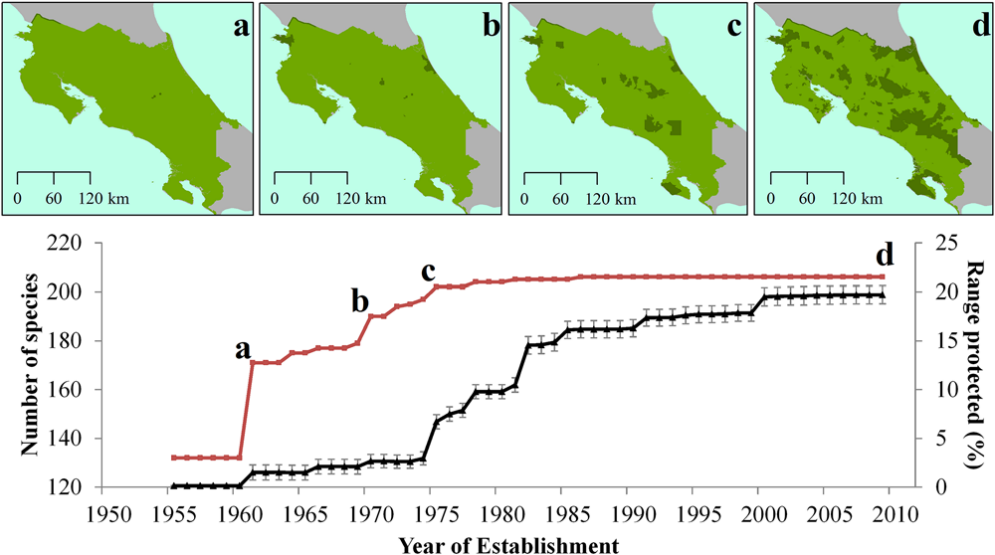
Chronology of protected areas establishment and number of mammal species represented in protected areas (red line), and mean percentage species ranges within protected areas (black line ± SE) in Costa Rica. Inset maps denote locations of protected areas for each major increase in number of species protected in a) 1960, b) 1970, c) 1975 and d) 2009 as also indicated in the graph.

**Fig 2:**
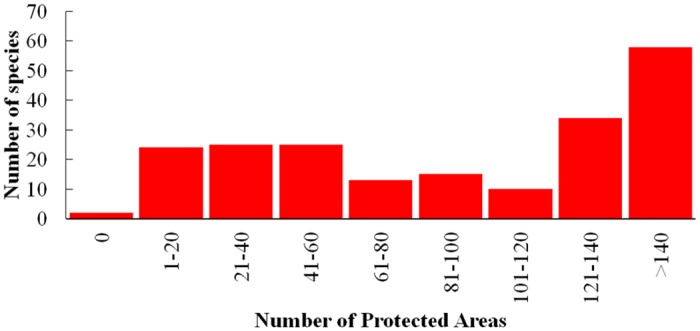
Frequency distribution of mammal species represented in all protected areas of Costa Rica. Note that most species were represented in at least 1 protected area and 48% of all species (100 species) were represented in more than 100 protected areas.

**Fig 3:**
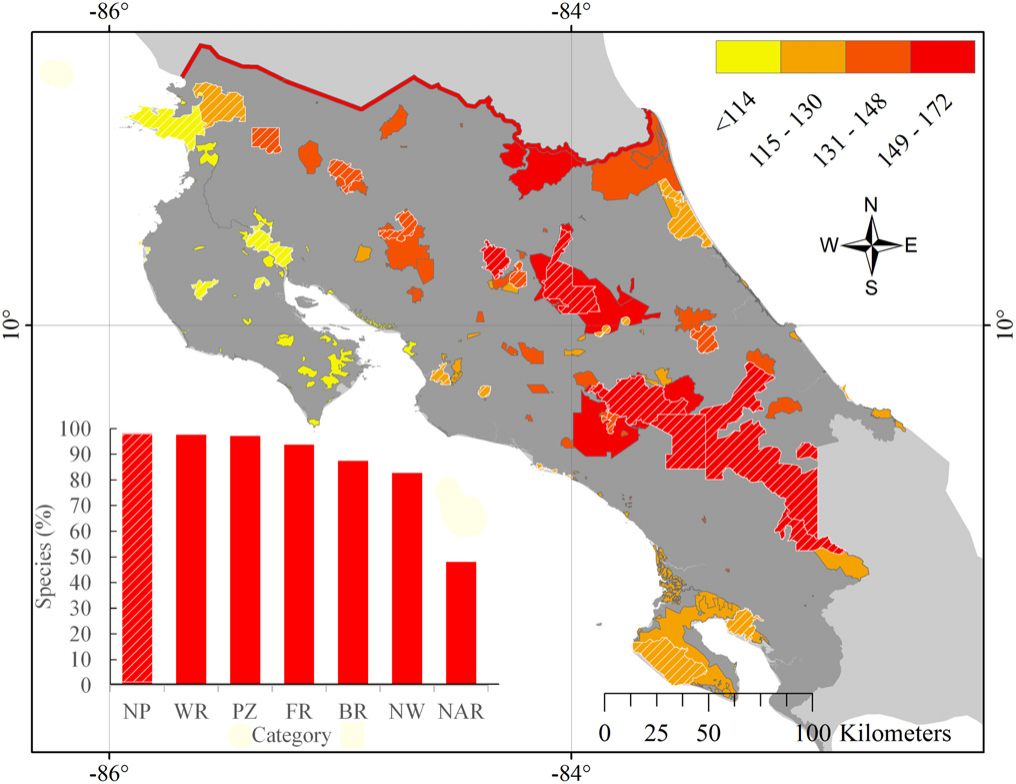
Number of mammal species by protected area and category in Costa Rica. The color scale at the top right indicates the number of species on each protected area. Protected areas with diagonal lines are National Parks. The inset figure indicates the percentage of mammal species in each protected area category (National Parks (NP), Wildlife Refuges (WR), Protected Zones (PZ), Forest Reserves (FR), Biological Reserves (BR), National Wetlands (NW) and Natural Absolute Reserves (NAR)).

About 82% of mammal species were represented in at least one protected area by 1961 and 97% were represented by 1975 (Fig 1). In 2014, the mean number of species per protected area is 123 (± 19) species (Median = 126), with the maximum number of species (*n* = 172) in La Amistad National Park and a minimum number of species (*n* = 93) in Costa Esmeralda Wildlife Refuge. More than 100 species are represented in >100 protected areas and 58 species in >140 protected areas; 76 species are protected in <60 protected areas (Fig 2). Although there is a significant positive relationship between the size of protected areas and the number of species, only 26% of the variation in the number of protected species is explained by the area of the reserve (R^2^ = 0.26, p>0.0001). Considering protected areas categories, National Parks contain the greatest species richness (98.10%), followed by Wildlife Reserves (97.60%), while National Absolute Reserves protect the fewest species (48.10%; Fig 3). National Parks and Protected Zones protect all endemic species, while National Wetlands and Absolute Natural Reserves protect 4 and 0 species, respectively. Considering threatened species, all protected area categories include all Endangered species (2 species). National Parks and Protected Zones protect all Data Deficient species (7 species) and these protected areas, along with Wildlife Refuges, include all Vulnerable species (4 species).

**Fig 4:**
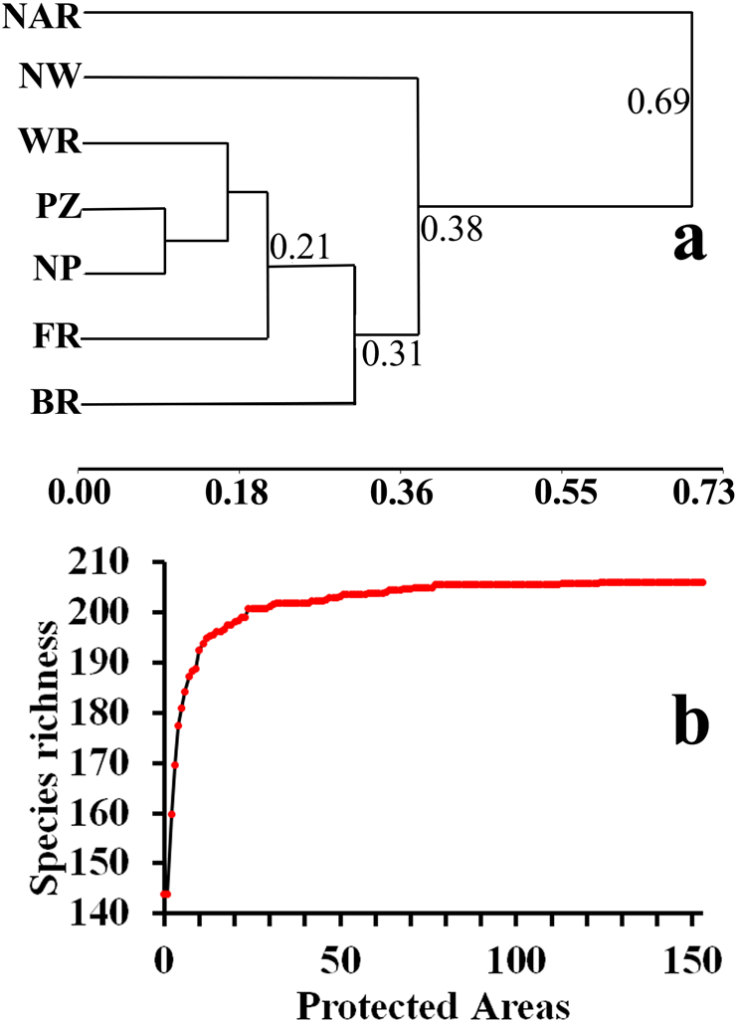
Complementarity of protected areas for representing mammal species richness in Costa Rica. a) Cluster analyses of species diversity using the Jaccard index between protected area categories (National parks (NP), Wildlife Refuges (WR), Forest Reserves (FR), Protected Zones (PZ), National Wetlands (NW) and Biological Reserves (BR)) and b) cumulative number of species protected.

We found low complementarity (i.e., high redundancy) among protected areas for representing mammal species, as evidenced by a mean Jaccard similarity coefficient of 0.68 (± 0.13). This complementarity was even lower within protected area categories, with a mean Jaccard coefficient of 0.80 (± 0.18). Diversity similarities regarding protected area categories indicated three main groups, where National Absolute Reserves and National Wetlands differed from each other and from the remaining categories (Jaccard coefficient = 0.69 and 0.38, respectively) and protected fewer species, while Protected Zones, National Parks, and Wildlife Refuges were similar and protect the greatest number of species (Fig 4a). Even when protected areas included a high overall percentage of mammal species richness, the total number of species protected was achieved with comparatively few protected areas (Fig 4b).

### Representativeness of mammal species ranges in protected areas

**Fig 5:**
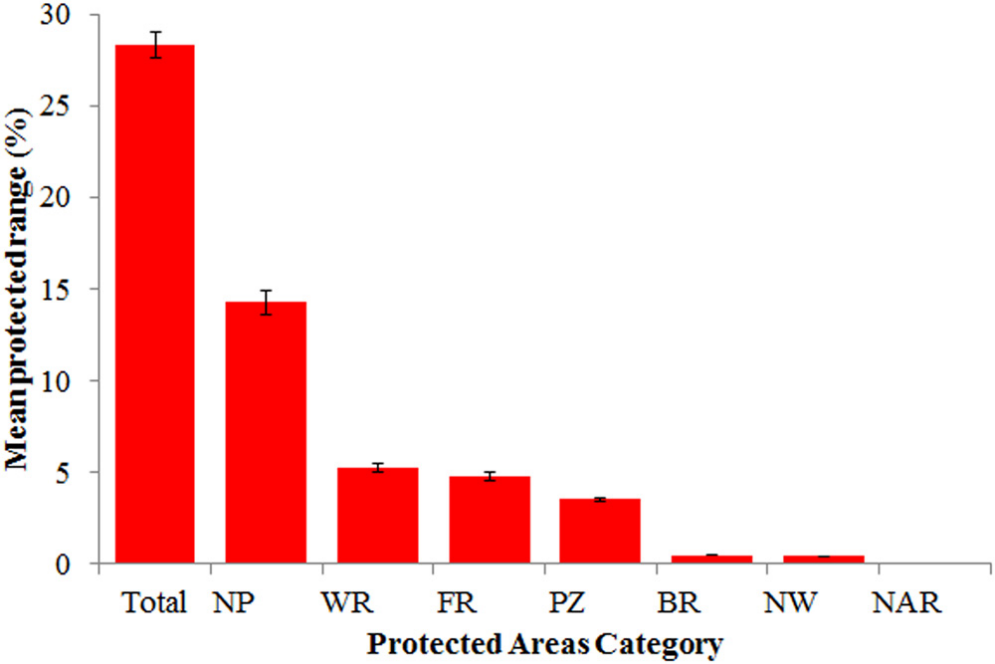
Percentage (±SE) of mammal species ranges within protected areas in Costa Rica. Mean percentage species ranges protected overall and in National Parks (NP), Wildlife Refuges (WR), Forest Reserves (FR), Protected Zones (PZ), Biological Reserves (BR), National Wetlands (NW) and National Absolute Reserves (NAR).

**Fig 6:**
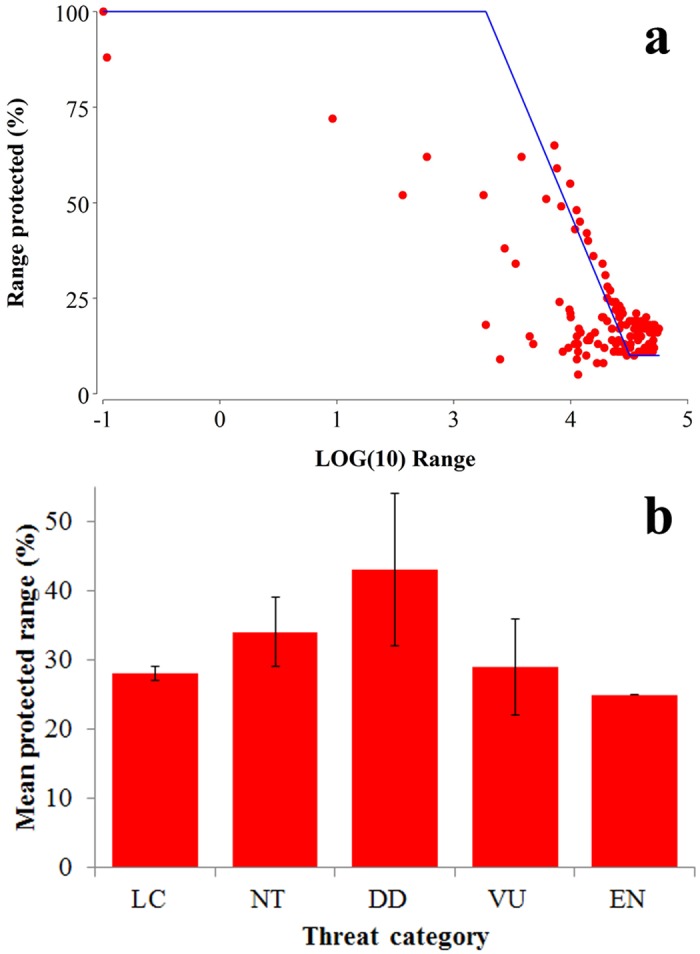
a) Mammal species range and percentage of each species range protected indicating a priori conservations targets (blue line) and b) percentage (±SE) of mammal species ranges protected in Costa Rica based on IUCN Red List of Threatened Species category (LC = Least Concern, NT = Near Threatened, DD = Data Deficient, VU = Vulnerable and EN = Endangered). Species located below the a priori target (see Methods) were identified as underprotected.

Though 98.50% of mammal species occur in at least one protected area, the geographic ranges of species protected within Costa Rica is considerably low. Considering all species, the mean percentage of protected range is 28.33 ± 13.85%, with National Parks protecting the greatest proportion of species ranges (14.27 ± 0.71%), followed by Wildlife Reserves (5.26 ± 0.66%). We found a strong correlation between the mean range of all species and the area covered by each category (*r* = 0.99, p <0.001). For four of the five remaining categories, mean percentage of species´ range protected was <5% (Fig 5). The temporal accumulation of mean species ranges within protected areas differed from that of the species richness. By 1960 only 1.50 ± 10.19% of the mean range of all mammals was protected, followed by 6.70 ± 10.40% by 1975 and reaching 19.72 ± 13.18% by 2009 (Median = 16.63%; Fig 1). Considering all species, 52 (25%) are underprotected in terms of range coverage by protected areas according to the a priori targets (Fig 6a). As for all endemic species, the overall mean range coverage is 38.70 ± 19.20%, with 10 endemic species under the proposed target (Table 2). Species classified as Data Deficient had on average 42.67 ± 27.55% of their geographic ranges protected, while the mean range protected for Vulnerable and Endangered species was 28.75 ± 13.32% and 25.00 ±0.01%, respectively (Fig 6b). Three species, *Cryptotis orophila* (DD), *Sigmodontomys aphrastus* (DD) and *Saimiri oerstedii* (VU) were considered underprotected according to our a priori targets. We found an inverse correlation between the total size of the species´ ranges in Costa Rica and the proportion protected (*r* = -0.76, p < 0.001).

### Conservation gaps and priority areas

**Fig 7:**
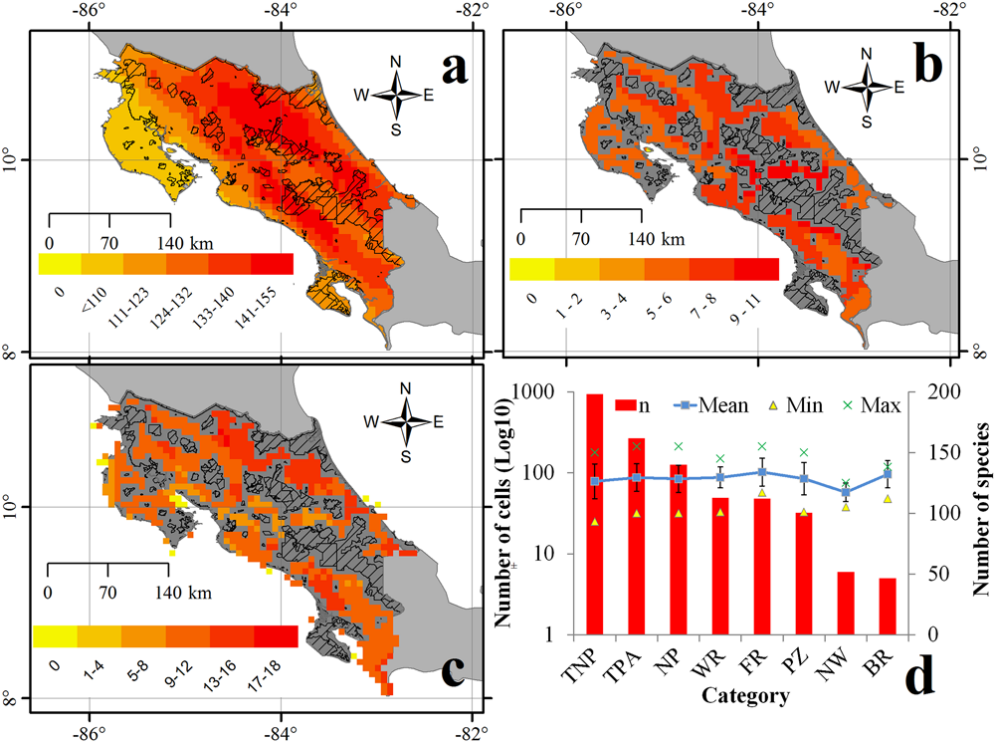
Mammal species richness within 83 km2 grid cells in Costa Rica and protected areas (dashed polygons). a) Total species richness; b) number of threatened (VU, EN, CR and DD) species in cells not covered by protected areas; c) number of underprotected species (according to a priori targets; see Methods) in cells not covered by protected areas, and; d) number of cells not protected (TNP), cells in all protected areas (TPA), and cells on each protected area category (National Parks (NP), Wildlife Refuges (WR), Forest Reserves (FR), Protected Zones (PZ), National Wetlands (NW) and Biological Reserves (BR)). Secondary Y axis indicates the mean (blue line + SD), minimum (triangles) and maximum (x) number of species protected by cell in each category.

Forty-three percent of cells are not currently included in any protected area. Mean species richness was significantly lower in cells without protected areas (121.41 ± 32.51) than in cells with ≥1 protected area (124.99 ±17.28; H = 5.51, p = 0.019). Unprotected areas include the Valle del General in the southern part of the country and the piedmont of the Tilaran Mountains in the north (Fig 7a). Also, a higher number of threatened species was estimated in cells with at least one protected area (7.38 ± 1.66) than in cells with none (6.47 ± 1.88; H = 42.94, p <0.0001; Fig 7b). A lower number of endemic species was estimated in unprotected (1.82 ± 2.38) than protected (3.16 ± 3.75) cells (H = 14.96, p < 0.001).

Priority areas to consider for filling gaps in range coverage for underprotected species (i.e., based on the a priori targets) were mostly in the Northern region and in the Valle del General of southern Costa Rica (Fig 7c). In general, most unprotected cells contained larger number of species, whereas Biological Reserves and National Wetlands contained the lowest number of species (Fig 7d).

## Discussion

Costa Rica´s protected areas system is one of the most extensive in Latin America, and is considered a management success [8], yet its representativeness and efficiency for biodiversity has been poorly studied. Protected areas contained 98.5% of terrestrial mammal species but we found low complementarity among these, with several areas contributing similarly to species richness and range coverage. A large proportion of species had substantial portions of their range protected, yet 25% of all species appear underprotected. Both endemic and threatened species were represented in at least a few protected areas, but species ranges were not adequately protected, especially for endemic species (10 species below the target).

Protected areas in Costa Rica contained at least a small percentage of the geographic ranges of most mammal species since the 1970s. The establishment of new areas since and up to the mid-1980s significantly increased mammal range coverage, not species, and remained fairly steady up to the year 2000 (i.e., about 19% mean range covered); since then new areas have not appreciably contributed to mammal protection nor markedly increased protection of areas with high species richness or mammal ranges. A large number of endemic species remain underprotected and require attention to reduce the potential for population losses. That many endemic species do not appear adequately protected suggests the approach used to design and establish protected areas has not incorporated all conservation needs that should be considered at a national scale to maximize biodiversity across ecosystems [33].

Complementarity is a key consideration when planning for conservation [32]. Since the contribution of different areas to the system is necessary to ensure representativeness, assessing this complementarity for Costa Rica could potentially inform planning for expanding and improving the current system. We found low complementarity (i.e. high redundancy) among protected areas for representing mammal species suggesting they have likely been established either opportunistically, without detailed information on species occurrence and distribution, or with emphasis on other taxonomic groups or different values (i.e. many National Parks were established around volcanoes), or even based on political will and agendas [18]. The high dependence of the Costa Rican NPAS on small reserves such as Wildlife Refuges (18% of all protected areas with a mean area of <26 km^2^), areas without complete protection (i.e., protected zones and forest reserves) and the lack of connectivity between them could compromise their potential for protecting many mammal populations and perhaps species. Thus, we highlight the importance of National Parks in protecting mammal diversity, as they include nearly 98.5% of all species in at least one area. Protection of endemic and threatened species is also of importance and highlights the need for systematic planning [33] that considers their protection. We note that although National Parks protected at least part of the range of almost all species, 25% of these species remain under the a priori target. Other categories of protected areas are generally in areas of the country least represented by National Parks and therefore could play an important role in protecting additional portions of species ranges.

In the absence of information regarding species population ecology, density or abundance, we considered range as the best available proxy for species´ populations, especially for an area as small as Costa Rica [5]. Conservation of wildlife, including mammals, is often based on conservation at the species and population levels [41], where population losses can adversely affect ecosystem function and services [5, 42]. We found for Costa Rica that at least 25% of all species are currently underprotected, a pattern similar to global analyses [6, 37]. However, the percentage of species underprotected in Costa Rica is greater than at global scale. More importantly, 55% of endemic and 23% of threatened species are currently underprotected, considerably greater than global values. This is of special concern for endemic species such as *Orthogeomys cherriei*, *O*. *heterodus* and *Reithrodontomys brevirostris*, which have a low percentage of their ranges currently protected (4.9, 9.1 and 12.3%, respectively). Protection of species richness depends on the extent of the protected area system as well as the range sizes of species. In Costa Rica, the relationship between range size and range protected was highly similar with global scales [37]; for species with small ranges, a general tendency to be either fully protected or drastically underprotected was observed, while species above our large range threshold (>25,550 km2) were well covered by the entire protected areas system.

The selection of potential additional areas for protection remains a complex task for Costa Rica. Considering the already extensive system in the country, few remaining areas appear to have largely intact ecosystems and are not currently dedicated to agriculture activities [11, 43, 44]. Nevertheless, these areas could represent a good investment by contributing to protecting ranges of underprotected species or species of conservation concern. Moreover, considering the low complementarity among categories, and the important role of small protected areas, conservation strategies could consider expanding current protected areas or ensuring connectivity corridors among these areas [45–47]. Previous assessments of gaps within the Costa Rican NPAS indicated that nearly 50% of life-zones were represented in only 2% of the protected areas, which highlights high biodiversity risks for these systems and the species that inhabit them [11]. Our approach incorporated not only the species presence but species range representativeness that can be used to emphasize potential risk for many species. For instance, special consideration should be given for species in the orders Carnivora, Didelphimorphia, Primates and Eulipotyphla considering the proportionally large number of threatened and declining species in these groups. Due to geographic extent constraints, few reserves in Costa Rica can likely harbor viable populations of many species, especially large mammals [48]. Therefore, incorporating species´ range into reserve planning or country-side conservation schemes [49] seems warranted to maintain mammal diversity in the country. Biological corridors have been identified as potential alternatives to protected areas in the country [45, 50], and thinking “outside the park” seems a necessary alternative to consider in light of potential mammal population losses [5, 49]. Already 26% of the country is under protection, but reserve size, isolation and anthropogenic pressures on these areas requires further evaluation of the protected areas system scheme [6, 14, 51]. Prioritization of expanded and connected reserve networks based on systematic conservation planning at landscape scales could help overcome the risks to perpetuate the representativeness of this protected areas system [52].

Costa Rica is an important area for continental mammal conservation [17, 24, 53, 54]; however, our results highlight the need for greater complementarity and representativeness [4, 55–58]. We have demonstrated the need for additional prioritization based on species and their geographic ranges, supporting the need for systematic conservation planning [59, 60]. The GRUAS II project (Technical proposal for territorial zoning for biodiversity conservation in Costa Rica) has identified several important areas for connectivity and species representation [61]. Though their analyses relied heavily on floristic components, several proposed areas were similar to areas we identified as important. Although in many respects highly successful, the Costa Rica NPAS would benefit from further protection schemes to ensure not only species richness, but species population viability, to better address the most serious threats in global biodiversity [5, 41].

We assessed the effectiveness of a protected areas system for one group of species. Our approach to identify protected area gaps in species and species’ range, including threatened and endemic species, could be applied to other taxa. Our results, together with assessments of other taxa could be used to help refine conservation priorities by incorporating additional levels of surrogacy and ecosystem level variations [62].

## Supporting Information

S1 Text Taxonomic information. (DOCX)

S1 Table List of Costa Rican mammal species used for analyzes classified by order and with its corresponding conservation status according to the IUCN Red List of Threatened Species. (DOCX)

S2 Table Species Richness, number of species on each category of the IUCN Red List of Threatened Species by taxa for Costa Rican mammals used for assessing their representativeness in protected areas. (DOCX)
