# Supplementary material for: Effectiveness of Protected Areas for Representing Species and Populations of Terrestrial Mammals in Costa Rica
Source: PLoS One. 2015 May 13;10(5):e0124480. doi: 10.1371/journal.pone.0124480 (PMC4430271; doi:10.1371/journal.pone.0124480)
Supplement: S1 Text — (DOCX) [file pone.0124480.s001.docx]

**S1 Text.** Taxonomic information

We used 208 species of mammals within 36 families and 11 orders (Table S1). The order with greatest number of species was Chiroptera (107 spp.) followed by Rodentia (50 spp.); Perissodactyla had the lowest species richness (Table S2). At the family level, Phyllostomidae (59 spp) had the greatest number of species followed by Cricetidae (31 spp); nine families contained only one species. Regarding conservation status, 185 species were considered Least Concern, 10 Near Threatened, 9 as Data Deficient and six were classified as threatened (4 Vulnerable and 2 Endangered). Population trends of 99 species were considered stable, 27 decreasing, 10 increasing and 74 as unknown. Perissodactyla, Carnivora and Cetartiodactyla were the orders with the greatest percentages of decreasing populations (100, 55 and 50% respectively), while Primates and Eulipotyphla have the greatest percentages of threatened species (50 and 25%, respectively; Table S2).

Taxonomic changes for the final list of species used in our analyses included our recognition of *Handleyomys alfaroi, Nephelomys devius* (endemic to Costa Rica and Panama)*, Sigmodontomys alfari, S. aphrastus, Transandinomys bolivaris,* and *T. talamancae* from the former *Oryzomys* genus following Weksler et al. [28]. We followed Musser and Carleton (1) for including former *Sigmodon hispidus* as *Sigmodon hirsutus*. *Spilogale angustifrons* was previously considered *S. putorius* [2]. Rodríguez-H, Chinchilla (3) excluded *Bassarycion pauli* and *B. lasius* as their taxonomy is unresolved, even though specimens were confirmed for the country and recent evidence suggests they all belong to *B. gabbii* [4]. Therefore, we retained only *B. gabbii* for analyses. For bats we recognized *Cynomops mexicanus* instead of *C. greenhalli* according to Peters, Lim (5), *Natalus stramineus* is now recognized as *N. mexicanus* [6], *Lonchophylla mordax* was included as *L. concava* [7], *Mimon cozumelae* was previously a subspecies of *M. benneti* and *Sturnira ludovici* is now used instead of *S. hondurensis* [6]. V*ampyressa pusilla* is now *V. thyone* [8] and *Cryptotis parva* is now considered *C. orophila* [9].

**References**

1. Musser GG, Carleton MD. Superfamily Muroidea. In: Wilson DE, Reeder DM, editors. Mammal Species of the world: a geographic and taxonomic reference. Baltimore, USA: The John Hopkins University Press; 2005. p. 894-1531.

2. Wozencraft WC. Order Carnivora. In: Wilson DE, Reeder DM, editors. Mammal Species of the World: a geographic and taxonomic reference. Baltimore, USA: The John Hopkins University; 2005. p. 532-628.

3. Rodríguez-H B, Chinchilla FA, May-Collado LJ. Lista de especies, endemismo y conservación de los de mamíferos de costa rica. Revista Mexicana de Mastozoología. 2002;6:21-57.

4. Helgen KM, Pinto M, Kays R, Helgen L, Tsuchiya M, Quinn A, et al. Taxonomic revision of the olingos (Bassaricyon), with description of a new species, the Olinguito. ZooKeys. 2013;324:1-83. doi: 10.3897/zookeys.324.5827.

5. Peters SL, Lim LK, Engstrom MD. Systematics of dog-faced bats (Cynomops) based on molecular and morphometric data. Journal of Mammalogy. 2002;83:1097-101.

6. Simmons N. Order Chiroptera. In: Wilson DE, Reeder DM, editors. Mammal Species of the World: a geographic and taxonomic reference. Baltimore, USA: The John Hopkins University; 2005. p. 312-529.

7. Albuja L, Gardner AL. A new species of *Lonchophylla* Thomas (Chiroptera: Phyllostomidae) from Ecuador. Proceedings of the Biological Society of Washington. 2005;118:442-9.

8. Lim BK, Pedro WA, Passos FC. Differentiation and species status of the Neotropical yellow-eared bats *Vampyressa pusilla* and *V. thyrone* (Phyllostomatidae) with a molecular phylogeny and review of the genus. Acta Chiroptera. 2003;5:15-29.

9. Woodman N. A new species of small-eared shrew from Colombia and Venezuela (Mammalia: Soricomorpha: Soricidae: Genus Cryptotis). Proceedings of the Biological Society of Washington 2002;115:249-72.
