# Supplementary material for: Effectiveness of Protected Areas for Representing Species and Populations of Terrestrial Mammals in Costa Rica
Source: PLoS One. 2015 May 13;10(5):e0124480. doi: 10.1371/journal.pone.0124480 (PMC4430271; doi:10.1371/journal.pone.0124480)
Supplement: S1 Table — (DOCX) [file pone.0124480.s002.docx]

**S1 Table**. List of Costa Rican mammal species used for analyzes classified by order and with its corresponding conservation status according to the IUCN Red List of Threatened Species. IUCN categories: Least Concern (LC), Near Threatened (NT), Data Deficient (DD), Vulnerable (VU) and Endangered (EN).

| **Order** | **Family** | **Species** | **IUCN Red List** | | | | |
| --- | --- | --- | --- | --- | --- | --- | --- |
|  |  |  | **LC** | **NT** | **DD** | **VU** | **EN** |
| Carnivora | Canidae | *Canis latrans* | X |  |  |  |  |
|  |  | *Urocyon cinereoargenteus* | X |  |  |  |  |
|  | Felidae | *Leopardus pardalis* | X |  |  |  |  |
|  |  | *Leopardus tigrinus* |  |  |  | X |  |
|  |  | *Leopardus wiedii* |  | X |  |  |  |
|  |  | *Panthera onca* |  | X |  |  |  |
|  |  | *Puma concolor* | X |  |  |  |  |
|  |  | *Puma yagouaroundi* | X |  |  |  |  |
|  | Mephitidae | *Conepatus semistriatus* | X |  |  |  |  |
|  |  | *Spilogale angustifrons* | X |  |  |  |  |
|  | Mustelidae | *Eira barbara* | X |  |  |  |  |
|  |  | *Galictis vittata* | X |  |  |  |  |
|  |  | *Lontra longicaudis* |  |  | X |  |  |
|  |  | *Mustela frenata* | X |  |  |  |  |
|  | Procyonidae | *Bassaricyon gabbii* | X |  |  |  |  |
|  |  | *Bassariscus sumichrasti* | X |  |  |  |  |
|  |  | *Nasua narica* | X |  |  |  |  |
|  |  | *Potos flavus* | X |  |  |  |  |
|  |  | *Procyon cancrivorus* | X |  |  |  |  |
|  |  | *Procyon lotor* | X |  |  |  |  |
| Cetartiodactyla | Cervidae | *Mazama temama* |  |  | X |  |  |
|  |  | *Odocoileus virginianus* | X |  |  |  |  |
|  | Tayassuidae | *Pecari tajacu* | X |  |  |  |  |
|  |  | *Tayassu pecari* |  | X |  |  |  |
| Chiroptera | Emballonuridae | *Balantiopteryx plicata* | X |  |  |  |  |
|  |  | *Centronycteris centralis* | X |  |  |  |  |
|  |  | *Cormura brevirostris* | X |  |  |  |  |
|  |  | *Cyttarops alecto* | X |  |  |  |  |
|  |  | *Diclidurus albus* | X |  |  |  |  |
|  |  | *Peropteryx kappleri* | X |  |  |  |  |
|  |  | *Peropteryx macrotis* | X |  |  |  |  |
|  |  | *Rhynchonycteris naso* | X |  |  |  |  |
|  |  | *Saccopteryx bilineata* | X |  |  |  |  |
|  |  | *Saccopteryx leptura* | X |  |  |  |  |
|  | Furipteridae | *Furipterus horrens* | X |  |  |  |  |
|  | Molossidae | *Cynomops mexicanus* | X |  |  |  |  |
|  |  | *Eumops auripendulus* | X |  |  |  |  |
|  |  | *Eumops glaucinus* | X |  |  |  |  |
|  |  | *Eumops hansae* | X |  |  |  |  |
|  |  | *Eumops underwoodi* | X |  |  |  |  |
|  |  | *Molossus coibensis* | X |  |  |  |  |
|  |  | *Molossus currentium* | X |  |  |  |  |
|  |  | *Molossus molossus* | X |  |  |  |  |
|  |  | *Molossus pretiosus* | X |  |  |  |  |
|  |  | *Molossus rufus* | X |  |  |  |  |
|  |  | *Molossus sinaloae* | X |  |  |  |  |
|  |  | *Tadarida brasiliensis* | X |  |  |  |  |
|  | Mormoopidae | *Pteronotus davyi* | X |  |  |  |  |
|  |  | *Pteronotus gymnonotus* | X |  |  |  |  |
|  |  | *Pteronotus parnellii* | X |  |  |  |  |
|  |  | *Pteronotus personatus* | X |  |  |  |  |
|  | Natalidae | *Natalus mexicanus* | X |  |  |  |  |
|  | Noctilionidae | *Noctilio albiventris* | X |  |  |  |  |
|  |  | *Noctilio leporinus* | X |  |  |  |  |

… Continue S1Table…

| **Order** | **Family** | **Species** | **LC** | **NT** | **DD** | **VU** | **EN** |
| --- | --- | --- | --- | --- | --- | --- | --- |
| Chiroptera | Phyllostomidae | *Anoura cultrata* |  | X |  |  |  |
|  |  | *Anoura geoffroyi* | X |  |  |  |  |
|  |  | *Artibeus aztecus* | X |  |  |  |  |
|  |  | *Artibeus jamaicensis* | X |  |  |  |  |
|  |  | *Artibeus lituratus* | X |  |  |  |  |
|  |  | *Artibeus phaeotis* | X |  |  |  |  |
|  |  | *Artibeus toltecus* | X |  |  |  |  |
|  |  | *Artibeus watsoni* | X |  |  |  |  |
|  |  | *Carollia castanea* | X |  |  |  |  |
|  |  | *Carollia perspicillata* | X |  |  |  |  |
|  |  | *Carollia sowelli* | X |  |  |  |  |
|  |  | *Carollia subrufa* | X |  |  |  |  |
|  |  | *Chiroderma salvini* | X |  |  |  |  |
|  |  | *Chiroderma trinitatum* | X |  |  |  |  |
|  |  | *Chiroderma villosum* | X |  |  |  |  |
|  |  | *Choeroniscus godmani* | X |  |  |  |  |
|  |  | *Chrotopterus auritus* | X |  |  |  |  |
|  |  | *Desmodus rotundus* | X |  |  |  |  |
|  |  | *Diaemus youngi* | X |  |  |  |  |
|  |  | *Diphylla ecaudata* | X |  |  |  |  |
|  |  | *Ectophylla alba* |  | X |  |  |  |
|  |  | *Enchisthenes hartii* | X |  |  |  |  |
|  |  | *Glossophaga commissarisi* | X |  |  |  |  |
|  |  | *Glossophaga leachii* | X |  |  |  |  |
|  |  | *Glossophaga soricina* | X |  |  |  |  |
|  |  | *Glyphonycteris sylvestris* | X |  |  |  |  |
|  |  | *Hylonycteris underwoodi* | X |  |  |  |  |
|  |  | *Lampronycteris brachyotis* | X |  |  |  |  |
|  |  | *Lichonycteris obscura* | X |  |  |  |  |
|  |  | *Lonchophylla concava* |  | X |  |  |  |
|  |  | *Lonchophylla robusta* | X |  |  |  |  |
|  |  | *Lonchorhina aurita* | X |  |  |  |  |
|  |  | *Lophostoma brasiliense* | X |  |  |  |  |
|  |  | *Lophostoma silvicolum* | X |  |  |  |  |
|  |  | *Macrophyllum macrophyllum* | X |  |  |  |  |
|  |  | *Mesophylla macconnelli* | X |  |  |  |  |
|  |  | *Micronycteris hirsuta* | X |  |  |  |  |
|  |  | *Micronycteris microtis* | X |  |  |  |  |
|  |  | *Micronycteris minuta* | X |  |  |  |  |
|  |  | *Micronycteris schmidtorum* | X |  |  |  |  |
|  |  | *Mimon cozumelae* | X |  |  |  |  |
|  |  | *Mimon crenulatum* | X |  |  |  |  |
|  |  | *Phylloderma stenops* | X |  |  |  |  |
|  |  | *Phyllostomus discolor* | X |  |  |  |  |
|  |  | *Phyllostomus hastatus* | X |  |  |  |  |
|  |  | *Platyrrhinus helleri* | X |  |  |  |  |
|  |  | *Platyrrhinus vittatus* | X |  |  |  |  |
|  |  | *Sturnira lilium* | X |  |  |  |  |
|  |  | *Sturnira ludovici* | X |  |  |  |  |
|  |  | *Sturnira luisi* | X |  |  |  |  |
|  |  | *Sturnira mordax* |  | X |  |  |  |
|  |  | *Tonatia saurophila* | X |  |  |  |  |
|  |  | *Trachops cirrhosus* | X |  |  |  |  |
|  |  | *Trinycteris nicefori* | X |  |  |  |  |
|  |  | *Uroderma bilobatum* | X |  |  |  |  |
|  |  | *Vampyressa nymphaea* | X |  |  |  |  |
|  |  | *Vampyressa thyone* | X |  |  |  |  |
|  |  | *Vampyrodes caraccioli* | X |  |  |  |  |
|  |  | *Vampyrum spectrum* |  | X |  |  |  |
|  | Thyropteridae | *Thyroptera discifera* | X |  |  |  |  |
|  |  | *Thyroptera tricolor* | X |  |  |  |  |

…Continue S1Table…

| **Order** | **Family** | **Species** | **LC** | **NT** | **DD** | **VU** | **EN** |
| --- | --- | --- | --- | --- | --- | --- | --- |
| Chiroptera | Vespertilionidae | *Bauerus dubiaquercus* |  | X |  |  |  |
|  |  | *Eptesicus brasiliensis* | X |  |  |  |  |
|  |  | *Eptesicus furinalis* | X |  |  |  |  |
|  |  | *Eptesicus fuscus* | X |  |  |  |  |
|  |  | *Lasiurus blossevillii* | X |  |  |  |  |
|  |  | *Lasiurus cinereus* | X |  |  |  |  |
|  |  | *Lasiurus ega* | X |  |  |  |  |
|  |  | *Lasiurus intermedius* | X |  |  |  |  |
|  |  | *Myotis albescens* | X |  |  |  |  |
|  |  | *Myotis elegans* | X |  |  |  |  |
|  |  | *Myotis keaysi* | X |  |  |  |  |
|  |  | *Myotis nigricans* | X |  |  |  |  |
|  |  | *Myotis oxyotus* | X |  |  |  |  |
|  |  | *Myotis riparius* | X |  |  |  |  |
|  |  | *Rhogeessa io* | X |  |  |  |  |
|  |  | *Rhogeessa tumida* | X |  |  |  |  |
| Cingulata | Dasypodidae | *Cabassous centralis* |  |  | X |  |  |
|  |  | *Dasypus novemcinctus* | X |  |  |  |  |
| Didelphimorphia | Didelphidae | *Caluromys derbianus* | X |  |  |  |  |
|  |  | *Chironectes minimus* | X |  |  |  |  |
|  |  | *Didelphis marsupialis* | X |  |  |  |  |
|  |  | *Didelphis virginiana* | X |  |  |  |  |
|  |  | *Marmosa mexicana* | X |  |  |  |  |
|  |  | *Metachirus nudicaudatus* | X |  |  |  |  |
|  |  | *Philander opossum* | X |  |  |  |  |
|  |  | *Micoureus alstoni* | X |  |  |  |  |
| Lagomorpha | Leporidae | *Sylvilagus brasiliensis* | X |  |  |  |  |
|  |  | *Sylvilagus dicei* |  |  | X |  |  |
|  |  | *Sylvilagus floridanus* | X |  |  |  |  |
| Perissodactyla | Tapiridae | *Tapirus bairdii* |  |  |  |  | X |
| Pilosa | Bradypodidae | *Bradypus variegatus* | X |  |  |  |  |
|  | Cyclopedidae | *Cyclopes didactylus* | X |  |  |  |  |
|  | Megalonychidae | *Choloepus hoffmanni* | X |  |  |  |  |
|  | Myrmecophagidae | *Myrmecophaga tridactyla* |  |  |  | X |  |
|  |  | *Tamandua mexicana* | X |  |  |  |  |
| Primates | Atelidae | *Alouatta palliata* | X |  |  |  |  |
|  |  | *Ateles geoffroyi* |  |  |  |  | X |
|  | Cebidae | *Cebus capucinus* | X |  |  |  |  |
|  |  | *Saimiri oerstedii* |  |  |  | X |  |

…Continue S1Table…

| **Order** | **Family** | **Species** | **LC** | **NT** | **DD** | **VU** | **EN** |
| --- | --- | --- | --- | --- | --- | --- | --- |
| Rodentia | Cricetidae | *Handleyomys alfaroi* | X |  |  |  |  |
|  |  | *Melanomys caliginosus* | X |  |  |  |  |
|  |  | *Nephelomys devius* | X |  |  |  |  |
|  |  | *Nyctomys sumichrasti* | X |  |  |  |  |
|  |  | *Oecomys trinitatis* | X |  |  |  |  |
|  |  | *Oligoryzomys fulvescens* | X |  |  |  |  |
|  |  | *Oligoryzomys vegetus* | X |  |  |  |  |
|  |  | *Oryzomys couesi* | X |  |  |  |  |
|  |  | *Ototylomys phyllotis* | X |  |  |  |  |
|  |  | *Peromyscus gymnotis* | X |  |  |  |  |
|  |  | *Peromyscus mexicanus* | X |  |  |  |  |
|  |  | *Reithrodontomys brevirostris* | X |  |  |  |  |
|  |  | *Reithrodontomys creper* | X |  |  |  |  |
|  |  | *Reithrodontomys fulvescens* | X |  |  |  |  |
|  |  | *Reithrodontomys gracilis* | X |  |  |  |  |
|  |  | *Reithrodontomys mexicanus* | X |  |  |  |  |
|  |  | *Reithrodontomys paradoxus* |  |  | X |  |  |
|  |  | *Reithrodontomys rodriguezi* | X |  |  |  |  |
|  |  | *Reithrodontomys sumichrasti* | X |  |  |  |  |
|  |  | *Rheomys raptor* | X |  |  |  |  |
|  |  | *Rheomys underwoodi* | X |  |  |  |  |
|  |  | *Scotinomys teguina* | X |  |  |  |  |
|  |  | *Scotinomys xerampelinus* | X |  |  |  |  |
|  |  | *Sigmodon hirsutus* | X |  |  |  |  |
|  |  | *Sigmodontomys alfari* | X |  |  |  |  |
|  |  | *Sigmodontomys aphrastus* |  |  | X |  |  |
|  |  | *Transandinomys bolivaris* | X |  |  |  |  |
|  |  | *Transandinomys talamancae* | X |  |  |  |  |
|  |  | *Tylomys nudicaudus* | X |  |  |  |  |
|  |  | *Tylomys watsoni* | X |  |  |  |  |
|  |  | *Zygodontomys brevicauda* | X |  |  |  |  |
|  | Cuniculidae | *Cuniculus paca* | X |  |  |  |  |
|  | Dasyproctidae | *Dasyprocta punctata* | X |  |  |  |  |
|  | Echimyidae | *Hoplomys gymnurus* | X |  |  |  |  |
|  |  | *Proechimys semispinosus* | X |  |  |  |  |
|  | Erethizontidae | *Coendou rothschildi* | X |  |  |  |  |
|  |  | *Sphiggurus mexicanus* | X |  |  |  |  |
|  | Geomyidae | *Orthogeomys cavator* | X |  |  |  |  |
|  |  | *Orthogeomys cherriei* | X |  |  |  |  |
|  |  | *Orthogeomys heterodus* | X |  |  |  |  |
|  |  | *Orthogeomys underwoodi* | X |  |  |  |  |
|  | Heteromyidae | *Heteromys desmarestianus* | X |  |  |  |  |
|  |  | *Heteromys oresterus* | X |  |  |  |  |
|  |  | *Liomys salvini* | X |  |  |  |  |
|  | Muridae | *Mus musculus* | X |  |  |  |  |
|  | Sciuridae | *Microsciurus alfari* | X |  |  |  |  |
|  |  | *Sciurus deppei* | X |  |  |  |  |
|  |  | *Sciurus granatensis* | X |  |  |  |  |
|  |  | *Sciurus variegatoides* | X |  |  |  |  |
|  |  | *Syntheosciurus brochus* |  | X |  |  |  |
| Soricomorpha | Soricidae | *Cryptotis gracilis* |  |  |  | X |  |
|  |  | *Cryptotis merriami* | X |  |  |  |  |
|  |  | *Cryptotis nigrescens* | X |  |  |  |  |
|  |  | *Cryptotis orophila* |  |  | X |  |  |
