# Supplementary material for: Effectiveness of Protected Areas for Representing Species and Populations of Terrestrial Mammals in Costa Rica
Source: PLoS One. 2015 May 13;10(5):e0124480. doi: 10.1371/journal.pone.0124480 (PMC4430271; doi:10.1371/journal.pone.0124480)
Supplement: S2 Table — (DOCX) [file pone.0124480.s003.docx]

**S2 Table.** Species Richness, number of species on each category of the IUCN Red List of Threatened Species by taxa for Costa Rican mammals used for assessing their representativeness in protected areas. IUCN categories: EN = Endangered, VU = Vulnerable, NT = Near Threatened, LC = Least Concern, and DD = Data Deficient.

| **Taxa** | **Species Richness** | **IUCN Red List** | | | | |
| --- | --- | --- | --- | --- | --- | --- |
|  |  | **LC** | **NT** | **DD** | **VU** | **EN** |
| **Carnivora** | **20** | **16** | **2** | **1** | **1** |  |
| Canidae | 2 | 2 |  |  |  |  |
| Felidae | 6 | 3 | 2 |  | 1 |  |
| Mephitidae | 2 | 2 |  |  |  |  |
| Mustelidae | 4 | 3 |  | 1 |  |  |
| Procyonidae | 6 | 6 |  |  |  |  |
| **Cetartiodactyla** | **4** | **2** | **1** | **1** |  |  |
| Cervidae | 2 | 1 |  | 1 |  |  |
| Tayassuidae | 2 | 1 | 1 |  |  |  |
| **Chiroptera** | **107** | **101** | **6** |  |  |  |
| Emballonuridae | 10 | 10 |  |  |  |  |
| Furipteridae | 1 | 1 |  |  |  |  |
| Molossidae | 12 | 12 |  |  |  |  |
| Mormoopidae | 4 | 4 |  |  |  |  |
| Natalidae | 1 | 1 |  |  |  |  |
| Noctilionidae | 2 | 2 |  |  |  |  |
| Phyllostomidae | 59 | 54 | 5 |  |  |  |
| Thyropteridae | 2 | 2 |  |  |  |  |
| Vespertilionidae | 16 | 15 | 1 |  |  |  |
| **Cingulata** | **2** | **1** |  | **1** |  |  |
| Dasypodidae | 2 | 1 |  | 1 |  |  |
| **Didelphimorphia** | **8** | **8** |  |  |  |  |
| Didelphidae | 8 | 8 |  |  |  |  |
| **Eulipotyphla** | **4** | **2** |  | **1** | **1** |  |
| Soricidae | 4 | 2 |  | 1 | 1 |  |
| **Lagomorpha** | **3** | **2** |  | **1** |  |  |
| Leporidae | 3 | 2 |  | 1 |  |  |
| **Perissodactyla** | **1** |  |  |  |  | **1** |
| Tapiridae | 1 |  |  |  |  | 1 |
| **Pilosa** | **5** | **4** |  |  | **1** |  |
| Bradypodidae | 1 | 1 |  |  |  |  |
| Cyclopedidae | 1 | 1 |  |  |  |  |
| Megalonychidae | 1 | 1 |  |  |  |  |
| Myrmecophagidae | 2 | 1 |  |  | 1 |  |
| **Primates** | **4** | **2** |  |  | **1** | **1** |
| Atelidae | 2 | 1 |  |  |  | 1 |
| Cebidae | 2 | 1 |  |  | 1 |  |
| **Rodentia** | **50** | **47** | **1** | **2** |  |  |
| Cricetidae | 31 | 29 |  | 2 |  |  |
| Cuniculidae | 1 | 1 |  |  |  |  |
| Dasyproctidae | 1 | 1 |  |  |  |  |
| Echimyidae | 2 | 2 |  |  |  |  |
| Erethizontidae | 2 | 2 |  |  |  |  |
| Geomyidae | 4 | 4 |  |  |  |  |
| Heteromyidae | 3 | 3 |  |  |  |  |
| Muridae | 1 | 1 |  |  |  |  |
| Sciuridae | 5 | 4 | 1 |  |  |  |
| **Country Total** | **208** | **185** | **10** | **7** | **4** | **2** |
